# Structure of anhydrotetracycline-bound Tet(X6) reveals the mechanism for inhibition of type 1 tetracycline destructases

**DOI:** 10.1038/s42003-023-04792-4

**Published:** 2023-04-17

**Authors:** Hirdesh Kumar, Emily E. Williford, Kevin S. Blake, Brett Virgin-Downey, Gautam Dantas, Timothy A. Wencewicz, Niraj H. Tolia

**Affiliations:** 1 Host-pathogen interaction and structural vaccinology section (HPISV), National Institute of Allergy and Infectious Diseases (NIAID), National Institutes of Health (NIH), Bethesda, MD USA; 2 Department of Chemistry, Washington University in St. Louis, One Brookings Drive, St. Louis, MO 63130 USA; 3 The Edison Family Center for Genome Sciences and Systems Biology, Washington University School of Medicine, St. Louis, MO USA; 4 Department of Pathology and Immunology, Division of Laboratory and Genomic Medicine, Washington University School of Medicine, St. Louis, MO USA; 5 Department of Molecular Microbiology, Washington University School of Medicine, St. Louis, MO USA; 6 Department of Biomedical Engineering, Washington University in St. Louis, St. Louis, MO USA; 7 Department of Pediatrics, Washington University School of Medicine, St. Louis, MO USA

**Keywords:** X-ray crystallography, Bacterial structural biology

## Abstract

Inactivation of tetracycline antibiotics by tetracycline destructases (TDases) remains a clinical and agricultural threat. TDases can be classified as type 1 Tet(X)-like TDases and type 2 soil-derived TDases. Type 1 TDases are widely identified in clinical pathogens. A combination therapy of tetracycline and a TDase inhibitor is much needed to rescue the clinical efficacy of tetracyclines. Anhydrotetracycline is a pan-TDase inhibitor that inhibits both type 1 and type 2 TDases. Here, we present structural, biochemical, and phenotypic evidence that anhydrotetracycline binds in a substrate-like orientation and competitively inhibits the type 1 TDase Tet(X6) to rescue tetracycline antibiotic activity as a sacrificial substrate. Anhydrotetracycline interacting residues of Tet(X6) are conserved within type 1 TDases, indicating a conserved binding mode and mechanism of inhibition. This mode of binding and inhibition is distinct from anhydrotetracycline’s inhibition of type 2 TDases. This study forms the framework for development of next-generation therapies to counteract enzymatic tetracycline resistance.

## Abstract (web summary)

Structural and biochemical characterization reveals that anhydrotetracycline’s mode of binding and inhibition of type 1 tetracycline destructases (TDases) is distinct from type 2 TDases.

## Introduction

Tetracyclines are a class of broad-spectrum antibiotics widely used in clinical and agricultural settings, and considered one of the big four antibiotics for human use^1^. Intensive use for more than eight decades has given rise to high abundance and diversity of tetracycline resistance genes in clinical pathogens. Historically, the two main mechanisms of tetracycline resistance have been ribosomal protection and drug efflux^2^. To maintain the efficacy of this drug class against antibiotic resistance by these mechanisms, third-generation tetracyclines have been recently developed by chemical modification of the tetracycline moiety^3–5^. Of these third-generation drug molecules, tigecycline is a last resort antibiotic used to treat infections with multidrug resistant (MDR) gram-negative bacteria and extensively drug-resistant (XDR) *Enterobacteriaceae* and *Acinetobacter* species^6,7^.

Although tigecycline and other last-generation tetracyclines circumvent resistance by ribosomal protection or efflux pumps, these molecules are inactivated by a group of enzymes called tetracycline destructases (TDases)^8,9^. In recent years, we and others have identified and characterized a plethora of TDases found in commensal, environmental, and pathogenic bacteria^10–15^. TDases are class A flavin-dependent monooxygenases that covalently modify and inactivate the core tetracycline moiety. Enzymatic inactivation of tetracyclines is unique in comparison to canonical tetracycline-resistance mechanisms because inactivation renders the tetracycline molecule incapable of further activity. TDases are broadly classified into two types based on sequence-structure-function characteristics: Tet(X)-like TDases and soil-derived TDases. Tet(X)-like TDases, which include the prototypical Tet(X) enzyme, have been identified in human gut metagenomes and pathogens, and can inactivate tetracyclines of all generations, including tigecycline and recently FDA-approved drugs sarecycline, eravacycline and omadacycline^10,12^. In contrast, soil-derived TDases have been primarily identified in soil metagenomes, and can inactivate first- and second- generation tetracyclines, but show limited activity against last-generation tetracyclines^16^. However, this soil-derived group of TDases can also be identified from other ecosystems. Tet(X)-like TDases and soil-derived TDases share only ~20% amino acid similarity and they have broadly similar structures^12,14,17,18^. Therefore, we propose naming these two classes of TDases as type 1 TDases for Tet(X)-like TDases; and type 2 TDases for those originally identified in soil metagenomes. A potent TDase inhibitor is needed for use in combination therapy to rescue the efficacy of the tetracycline group of antibiotics against pathogens expressing TDases.

Anhydrotetracycline (aTC) is the first broad spectrum TDase inhibitor that blocks type 1 and type 2 TDases in vitro and in bacterial phenotypic assays^12^. We previously reported the crystal structure of anhydrotetracycline bound to the type 2 TDase Tet(50), showing it is a competitive inhibitor that binds in a distinct mode in the substrate binding cavity^12^; these structural insights enabled development of anhydrotetracycline derivatives as additional type 2 TDase inhibitors^19^. However, the binding and inhibition mode of anhydrotetracycline to type 1 TDases are unknown.

Here, we investigate Tet(X6), a type 1 TDase first discovered in a *Proteus* genomospecies^20^, by determining anhydrotetracycline-free and anhydrotetracycline-complexed X-ray crystal structures. Tet(X6) inactivates all classes of tetracyclines demonstrated by in vitro enzyme assays and phenotypic studies in *E. coli*. The whole cell growth inhibitory activity of tetracycline against *E. coli* expressing Tet(X6) is rescuable with anhydrotetracycline. The structures revealed that anhydrotetracycline binds in a substrate-like orientation in Tet(X6) and serves as a competitive inhibitor. In contrast, type 2 TDases are unable to metabolize anhydrotetracycline, and anhydrotetracycline binds to type 2 TDases in a distinct orientation as a mechanistic and competitive inhibitor^12^. Michaelis-Menten steady-state kinetic studies confirmed that anhydrotetracycline is a substrate for Tet(X6). Direct detection of the anhydrotetracycline Tet(X6) oxidation product by LC-MS further supports a model for competitive inhibition as a sacrificial substrate. The current study explains the differences in structural and functional characteristics of anhydrotetracycline between the two classes of TDases.

## Results

### Tet(X6) contains a conserved architecture of type 1 tetracycline destructases

**Fig. 1:**
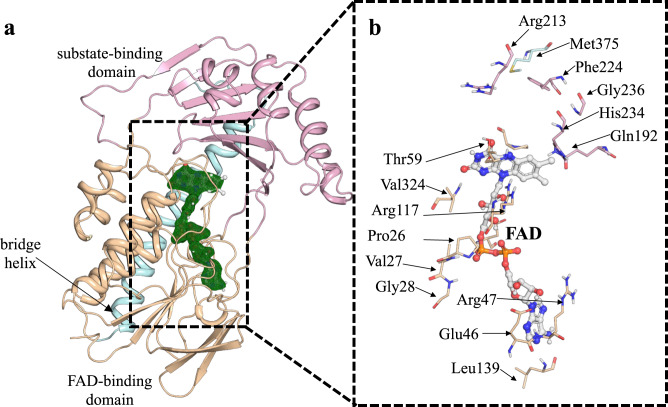
Tet(X6) has a conserved architecture of type 1 TDases. **a** Structure of anhydrotetracycline free Tet(X6) determined here (8ER1). The substrate-binding domain is colored pink. The FAD-binding domain is colored orange. The C-terminal bridge helix is colored blue. Omit map for the bound FAD omitted from the Tet(X6) anhydrotetracycline free structure determined here (8ER1) is shown in green and density is contoured at 2.5 σ. The FAD is bound in an “IN” orientation consistent with previously solved type 1 TDase structures. **b** Key residues in the Tet(X6) anhydrotetracycline free structure determined here (8ER1) that interact with FAD are shown. Residues in the substrate binding pocket are also highlighted. Color scheme: orange – residues from the FAD binding domain; pink – residues from the substrate binding domain; and blue – residues from the C-terminal bridge helix.

We determined the X-ray structure of Tet(X6) at 2.2 Å resolution with Rfree/Rwork of 0.22/0.19 (Table 1). This FAD complexed, anhydrotetracycline-free enzyme structure consists of a conserved architecture for type 1 TDases: a characteristic Rossman fold containing an FAD-binding domain, a substrate binding domain, and a C-terminal bridge helix that connects the two domains (Fig. 1). The 393 amino acid long polypeptide chain alternates four times between the substrate-binding domain and FAD-binding domain. The Tet(X6) holo-enzyme resembles previously reported type 1 TDases with high structural similarity of <0.5 Å r.m.s.d. on C^ɑ^ atoms, and high sequence identity of >85% (Supplementary Fig. 1)^14,17,18^.

**Table 1:** Data Reduction Statistics for the Tet(X6) and Tet(X6) in complex with anhydrotetracycline structures.

	Tet(X6) (8ER1)	Tet(X6) + Anhydrotetracycline (8ER0)
Data collection		
Space group	P 1 21 1	P 1 21 1
Cell dimensions		
* a*, *b*, *c* (Å)	43.77, 52.49, 95.17	87.09, 52.21, 94.87
α, β, γ (°)	90, 95.88, 90	90, 95.44, 90
Resolution (Å)	19.9–1.9 (1.94–1.9)	19.84–2.2 (2.28–2.2)
* R*_merge_	0.031 (0.234)	0.095 (0.592)
* I* / σ*I*	18.1 (2.8)	9.8 (5.1)
Completeness (%)	98.8 (98.0)	98.8 (98.0)
Redundancy	3.5 (3.3)	3.5 (3.4)
Refinement		
Resolution (Å)	19.9–1.9 (1.94–1.9)	19.84–2.2 (2.28–2.2)
No. reflections	33,536 (3273)	42,923 (4215)
* R*_work_ / *R*_free_	0.20/0.23	0.19/0.23
No. atoms		
Protein	2952	5877
Ligands	84	268
Water	152	263
* B*-factors		
Protein	41.1	33.1
Ligands	33.6	38.7
Water	41.8	32.9
R.m.s. deviations		
Bond lengths (Å)	0.011	0.003
Bond angles (°)	1.09	0.57

Each structure was solved from a single crystal. Values in parentheses are for highest-resolution shell.

Tet(X6) contains a single C-terminal bridge helix similar to other type 1 TDase structures (Tet(X2) – PDB 2Y6R, Tet(X4) – PDB 7EPV, and Tet(X7) – PDB 6WG9) (Supplementary Fig. 1) and this constitutes a major distinction from type 2 TDases that contain a 2nd ɑ-helix at the C-terminus^12^ (Fig. 1 and Supplementary Fig. 2). The FAD cofactor is bound in an “IN” orientation in Tet(X6). Thus far, the FAD conformation in structures of type 1 TDases have only been captured in this “IN” orientation, while structures of type 2 TDases have been solved for both “IN” and “OUT” orientations. It is assumed, but not yet experimentally validated, that the FAD cofactor present in the type 1 TDases share this two-state binding mode. The FAD-binding pocket and the substrate-binding pocket are conserved among all four of the solved type 1 TDase structures (Fig. 1 and Supplementary Fig. 1). The conserved FAD-interacting residues include Pro26, Val27, Gly28, Glu46, Arg47, Thr59, Arg117, Leu139, and Val324. The conserved substrate-binding residues are Gln192, Arg213, Phe224, His234 and Gly236. Met375 of the C-terminal bridge helix also interacts with the bound tetracycline substrate and is conserved among the type 1 TDases (Fig. 1 and Supplementary Figs. 1,  3). The asymmetric units and packing of crystal lattices of Tet(X6) X-ray crystal structures are shown in Supplementary Fig. 4. Despite structural similarities, type 1 TDases can show significant differences in enzyme kinetics with up to ~10-fold difference in apparent catalytic efficiency^14^. Mutations outside the TDase active site generated via directed evolution and natural selection have been shown to enhance enzyme efficiency and resistance levels in whole cell assays^17,21^. It is likely that surface-exposed residues affect both conformational dynamics and active site environments of different TDases, leading to differences in protein stability and enzyme efficiency that influence resistance phenotypes. Additional putative binding sites have been observed at the entrance of the active site in the Tet(X)-minocycline complex crystal structure (PDB ID: 4A99) that could plausibly guide the substrate into the active site^22^.

### Tet(X6) confers pan-tetracycline-resistance

**Fig. 2:**
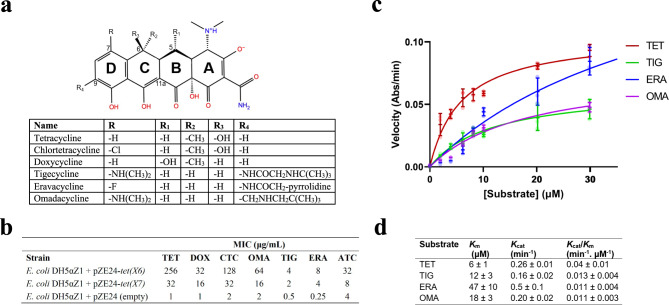
Tet(X6) confers pan tetracycline resistance. **a** Chemical structures of different tetracyclines. **b** Tet(X6) confers high minimum inhibitory concentrations (MIC). Abbreviations: tetracycline (TET); doxycycline (DOX); chlortetracycline (CTC); omadacycline (OMA); tigecycline (TIG); eravacycline (ERA); anhydrotetracycline (aTC). **c** Michaelis-Menten curves for Tet(X6)-catalyzed degradation of tetracycline antibiotics. Note that the 65 µM data point for eravacycline is omitted for uniform scale on the *x*-axis and the full plot is provided as Supplementary Fig. 5. **d** Apparent *K*_m_, *k*_cat_, and catalytic efficiencies (*k*_cat_/*K*_m_) of Tet(X6) against different tetracyclines. Error values represent standard deviations for three independent trials.

We validated the activity of Tet(X6) in the *E. coli* DH5αZ1 + pZE24 system using microbroth dilution antibiotic susceptibility tests^23^ (Fig. 2a, b). For positive and negative controls, we also characterized the resistance profiles of *E. coli* strains producing Tet(X7) or containing the empty pZE24 vector (*i.e*., with no tetracycline resistance gene). In accordance with the initial report on this enzyme^20^, heterologous expression of Tet(X6) in *E. coli* conferred high minimal inhibitory concentrations (MICs) against tetracycline antibiotics from all three generations (Fig. 2b). The highest MICs were against first-generation drug molecules tetracycline and chlortetracycline (256 and 128 μg/mL, respectively). Consistent with other type 1 TDases, Tet(X6) also conferred resistance to doxycycline, a second-generation tetracycline, as well as third-generation tetracyclines tigecycline, omadacycline, and eravacycline^5,24^. Notably, the Tet(X6) strain’s MICs are 2- to 8-fold higher than the Tet(X7) strain, which had been previously considered one of the most active TDases^14,19^ (Fig. 2b).

To study substrate binding and catalytic efficiency of Tet(X6) under steady state conditions, we continuously monitored the change in absorbance at 400 nm (unique λ_max_ for tetracyclines) to observe direct enzymatic inactivation of tetracycline substrates: tetracycline, tigecycline, omadacycline, and eravacycline by Tet(X6) (Fig. 2c, d). The velocity versus substrate concentration curves for tetracycline and tigecycline were hyperbolic with good fit to the standard Michaelis-Menten equation. The curves for eravacycline and omadacycline appeared sigmoidal in nature, indicating the potential for allostery or multiple binding orientations for the substrate, the latter of which has been observed for the TDase family^12,18^. Curve fitting to Michaelis-Menten and allosteric sigmoidal models in GraphPad prism both produced acceptable fits (R^2^ > 0.95) (Supplementary Fig. 5; Supplementary Data 1). Based on the Michaelis-Menten fits for all antibiotic substrates, the range of apparent *K*_m_ values was 5–57 within the error of uncertainty ranging from 17–25% for these data fits (Fig. 2d). Among the five substrates studied, tetracycline showed the lowest apparent *K*_m_ value of 6 ± 1 µM, suggesting the highest apparent binding affinity, followed by tigecycline (apparent *K*_m_ = 12 ± 3 µM), omadacycline (apparent *K*_m_ = 18 ± 3 µM), and eravacycline (apparent *K*_m_ = 47 ± 10 µM). The apparent catalytic efficiency of Tet(X6) was greatest for tetracycline as the substrate (*k*_cat_/*K*_m_ = 0.04 ± 0.01 min^−1^µM^−1^) compared to third-generation tetracycline substrates, and is mainly driven by *K*_m_. The apparent catalytic efficiencies (*k*_cat_/*K*_m_) for Tet(X6)-catalyzed oxidation of eravacycline (*k*_cat_/*K*_m_ = 0.011 ± 0.004 min^−1^µM^−1^), tigecycline (*k*_cat_/*K*_m_ = 0.013 ± 0.004 min^−1^µM^−1^), and omadacycline (*k*_cat_/*K*_m_ = 0.011 ± 0.003 min^−1^µM^−1^) were similar.

### Anhydrotetracycline rescues Tet(X6)-mediated inactivation of tetracycline antibiotics

**Fig. 3:**
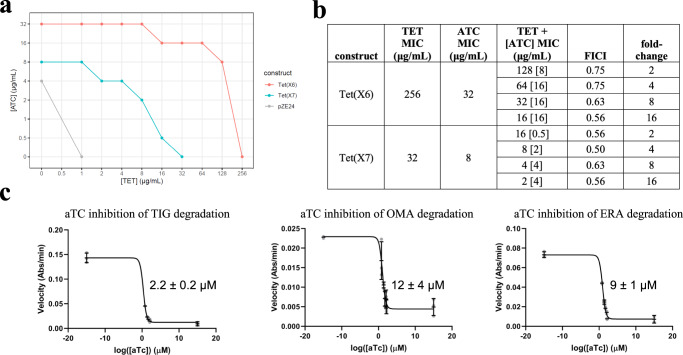
Anhydrotetracycline rescues Tet(X6)-mediated inactivation of tetracycline antibiotics. **a** Whole cell inhibition of *E. coli* expressing tetracycline destructase enzymes. Abbreviations: tetracycline (TET); anhydrotetracycline (aTC). **b** Calculated FICI. **c** In vitro aTC inhibition of Tet(X6) degradation of tetracycline antibiotics as observed via an optical absorbance assay. IC_50_ values of each curve are shown. Error values represent standard deviations for three independent trials.

We have previously established that anhydrotetracycline inhibits a wide range of TDases and therefore can be classified as a pan-TDase inhibitor^12,19^. Here, we study if anhydrotetracycline can rescue tetracycline activity against *E. coli* producing Tet(X6). We identified anhydrotetracycline concentrations that result in a tetracycline MIC lower than that of tetracycline alone, using checkerboard broth microdilution antibiotic susceptibility assays which test for cell growth in multiple tetracycline-anhydrotetracycline combinations. The addition of 16 μg/mL anhydrotetracycline reduced by 16-fold the concentration of tetracycline required to inhibit growth of Tet(X6)-producing *E. coli*, from 256 μg/mL to 16 μg/mL (Fig. 3a). The calculated fractional inhibitory concentration (FICI) index^25^ is provided for reference (Fig. 3b).

While anhydrotetracycline has antibiotic activity on its own against *E. coli*, we used concentrations below the MIC (32 μg/mL; Fig. 2b), then evaluated the inhibitory activity of anhydrotetracycline against Tet(X6)-mediated degradation of tetracycline antibiotics (Fig. 3c). The apparent half-maximal inhibitory concentrations (IC50s) observed were in the low micromolar range (2–12 µM), consistent with concentrations used in the whole cell rescue assays. Together these results suggest that, as with other TDases, anhydrotetracycline inhibition is a promising combination therapy against bacteria producing Tet(X6).

### Anhydrotetracycline binds in a substrate-like orientation in type 1 TDases

**Fig. 4:**
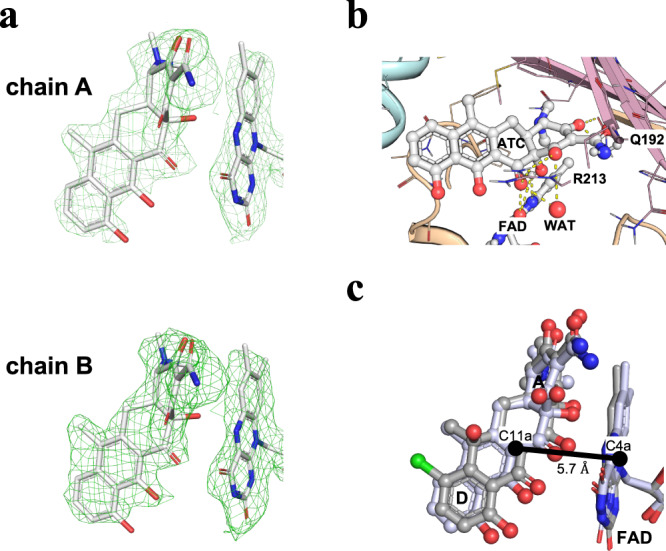
Anhydrotetracycline binds in a substrate-like orientation in Tet(X6). **a** Polder maps (Fo-Fc map contoured at 3 σ) identify the substate-like binding orientation of anhydrotetracycline (aTC) in the two chains of the Tet(X6) crystal structure. The complete Polder map is shown in Supplementary Fig. 6. Asymmetric units of anhydrotetracycline-complexed Tet(X6) X-ray crystal structure is shown in Supplementary Fig. 4. **b** Key interactions of anhydrotetracyclines with active site residues of Tet(X6). **c** Aligned crystal structures of Tet(X6)-anhydrotetracycline complex with Tet(X)-chlortetracycline (CTC) complex structure (PDB ID: 2Y6R). Aligned CTC and FAD from Tet(X) structures are shown in grey. The C11a of aTC is at 5.7Å from C4a of the FAD isoalloxazine ring.

An X-ray co-crystal structure of anhydrotetracycline in complex with Tet(X6) was determined at 2.2 Å resolution (Table 1). A clear non-protein density was observed in the substrate-binding cavity, consistent with the size and shape of anhydrotetracycline (Fig. 4a). This structure shows that anhydrotetracycline binds in a substrate-like orientation in Tet(X6). The isoalloxazine group of bound FAD occupies an ‘IN’ orientation in the Tet(X6)-anhydrotetracycline complex structure, as observed for the anhydrotetracycline-free structure (Fig. 4a). The anhydrotetracycline binds to Tet(X6) in a substrate-like orientation, placing the A-ring close to the FAD and the D-ring close to the C-terminal bridge helix. The orientation of anhydrotetracycline in Tet(X6) remains conserved in structures of tetracycline substrates in complex with other type 1 TDases, and is likely driven by the shared planarity of rings B, C, and D between anhydrotetracycline and other tetracycline substrates^14,17,18^.

To compare polar and non-polar interactions of anhydrotetracycline in Tet(X6) with previously studied interactions in other type 1 TDase-substrate complexes, we aligned the Tet(X6)-anhydrotetracycline complex structure to a previously solved X-ray crystal structure of Tet(X) in complex with chlortetracycline (PDB ID: 2Y6R). The protein-ligand interactions between the tetracycline moieties and the residues of type 1 TDases remain conserved in the Tet(X6)-anhydrotetracycline and Tet(X)-chlortetracycline structures^18^ (Supplementary Fig. 3). For example, the substitution of the 2,3-enol hydroxyl group at the A-ring of the bound anhydrotetracycline forms a hydrogen bond with side chains of Gln192 (Fig. 4b). The carboxamide carbonyl oxygen at the 2-position of anhydrotetracycline forms a hydrogen bond with Arg213. A water molecule serves as a hydrogen-bonding bridge between the carboxamide substitution of anhydrotetracline and Thr59. In addition, the Phe224-side chain stabilizes the bound anhydrotetracycline through π-cation interaction with the 7-dimethylamino substitution of anhydrotetracycline. Further, the isoalloxazine-ring system of FAD forms two additional H-bonds with anhydrotetracycline. The O4 and N5 atoms of FAD form H-bonds to the keto-enol moiety (O12) and nearby hydroxyl group (O12a) of bound anhydrotetracycline. These interactions contribute to orienting the anhydrotetracycline scaffold for hydroxylation by a putative C4a-peroxy-flavin reactive intermediate at site C11a, with at an appropriate short distance of ~5.6 Å from the C4a of FAD-isoalloxazine heterocycle. Anhydrotetracycline also makes two H-bonds with aromatic amino acids: hydrogen atom at the C-7 position in the D-ring of anhydrotetracycline with the backbone oxygen atom of Phe319; and oxygen of the carboxamide moiety attached to the C-2 position of the A-ring of anhydrotetracycline with the aromatic hydrogen atom of His234 (Fig. 4b). An alignment of the Tet(X6)-anhydrotetracycline complex with previously solved Tet(X)-chlortetracycline complex (PDB ID: 2Y6R) confirmed a conserved distance of ~5.7 Å between C11a of anhydrotetracycline and C4a of the FAD isoalloxazine ring, suggesting a correlation of this binding mode with hydroxylation of C11a across substrate classes (Fig. 4c).

### Anhydrotetracycline oxidation is catalyzed by type 1 TDases

**Fig. 5:**
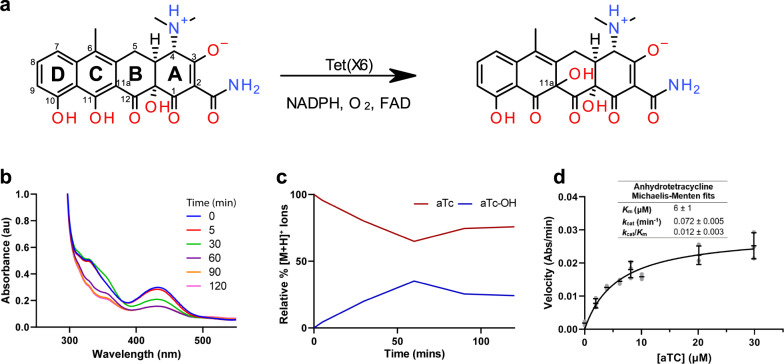
Anhydrotetracycline oxidation is catalyzed by type 1 TDases. **a** C11a is the plausible site of oxidation in aTC. **b** Degradation of aTC as observed via optical absorbance spectroscopy. **c** Extracted mass ion counts (normalized as relative %) for aTC and oxidized aTC-OH from LC-MS of Tet(X6) reaction from panel (**b**). **d** Michaelis-Menten steady-state kinetic curve for Tet(X6)-catalyzed degradation of aTC. Error values represent standard deviations for three independent trials.

We have previously established that anhydrotetracycline is a pan destructase inhibitor^10,12^ that inhibits diverse type 1 and type 2 TDases. While type 2 TDases cannot metabolize anhydrotetracycline, type 1 TDases such as Tet(X) are capable of slowly turning over anhydrotetracycline as a substrate. The Tet(X6)-anhydrotetracycline complex structure shows a substrate-like binding mode of anhydrotetracycline in Tet(X6). Therefore, we speculated that Tet(X6) can oxidize anhydrotetracycline at the C11a atom (Fig. 5a). To assess the potential for anhydrotetracycline to serve as a substrate for Tet(X6), we performed an in vitro optical absorbance kinetic assay and LC-MS analysis, as previously reported^12^. We observed degradation of anhydrotetracycline as indicated by the time-dependent decrease at 440 nm (unique λ_max_ for anhydrotetracycline under assay conditions) in the presence of Tet(X6), NADPH, and O_2_ (Fig. 5b). We analyzed the same reaction mixtures by LC-MS and identified ions corresponding to the predicted mass for the [M + O + H]^+^ molecular ion of oxidized anhydrotetracycline that appeared with stoichiometric loss of the [M + H]^+^ ion corresponding to the parent anhydrotetracycline predicted mass (Fig. 5c). To evaluate the catalytic efficiency of Tet(X6) degradation of anhydrotetracycline, Michaelis-Menten steady-state kinetics were determined (Fig. 5d). The observed apparent binding affinity (*K*_m_) was 6 ± 1 µM, similar to tetracycline, but lower than tigecycline (apparent *K*_m_ = 12 ± 3 µM), omadacycline (apparent *K*_m_ = 18 ± 3 µM), and eravacycline (apparent *K*_m_ = 47 ± 10 µM) (Fig. 2d). The apparent *k*_cat_ for Tet(X6) with anhydrotetracycline as the substrate, however, was significantly lower than tetracycline antibiotic substrates by up to a factor of 7, at 0.072 ± 0.005 µM (Fig. 5d). This implies that the reduced catalytic efficiency of Tet(X6) for turning over anhydrotetracycline (*k*_cat_/*K*_m_ = 0.012 ± 0.003 min^−1^µM^−1^) compared to tetracycline (*k*_cat_/*K*_m_ = 0.04 ± 0.01 min^−1^µM^−1^) is driven by an apparent reduction in *k*_cat_. This is consistent with the model that anhydrotetracycline binds rapidly in the active site of Tet(X6) and serves as a competitive substrate for Tet(X6) with a much lower rate of catalysis (*k*_cat_). The binding orientation and enzyme kinetics of anhydrotetracycline in Tet(X6) open new avenues for rational drug design to develop potent anhydrotetracyline-based type 1 TDase inhibitors that further reduce *k*_cat_ or eliminate catalysis.

## Discussion

In this study, we determined X-ray structures of the Type 1 TDase Tet(X6) as a *holo*-enzyme (FAD-bound) and in complex with anhydrotetracycline. The X-ray structure of the Tet(X6)-anhydrotetracycline complex demonstrates a substrate-like binding orientation of anhydrotetracycline, supported by biochemical and cellular studies which indicate a substrate-like metabolism. We established that anhydrotetracycline competitively inhibits Tet(X6)-mediated inactivation of tetracycline antibiotics despite the ability of Tet(X6) to turnover anhydrotetracycline as a substrate. This type of sacrificial substrate inhibition of enzymes serves as the basis of clinically useful β-lactamase inhibitor/ β-lactam antibiotic combination therapies^26–31^. The inhibition of β-lactamases is achieved through an initial hydrolysis of the bound inhibitor leading to covalent adduct formation (clavulanic acid and sulbactam) or reversible hydrolysis (avibactam). Here, the inhibition of Tet(X6) by anhydrotetracycline is reversible and the oxidized anhydrotetracycline product is released. Hence, the apparent inhibition of Tet(X6) arises due to the ability of anhydrotetracycline to outcompete tetracycline antibiotics for binding combined with a slower rate of enzymatic turnover of anhydrotetracycline relative to tetracyclines. The sacrificial nature of anhydrotetracycline occupies TDases in bacterial cells to reduce the rate and likelihood of TDase-mediated degradation of tetracycline antibiotics when used in combination therapies. The type 2 TDases cannot metabolize anhydrotetracycline in this manner^12^. Type 1 TDases from clinical pathogens contain conserved residues in the FAD-binding pocket and in the substrate-binding pocket, suggesting a shared mechanism of anhydrotetracycline binding and enzyme inhibition^14,17,18^. Our X-ray crystal structures provide insights into the binding mode of anhydrotetracycline in type 1 TDases and may guide design and development of more potent TDase inhibitors that do not act as sacrificial substrates^32^.

Multiple recent studies have suggested combination therapy consisting of a tetracycline antibiotic with a TDase inhibitor is also feasible^12,33–35^. Park et al. reported that anhydrotetracycline can rescue tetracycline efficacy in pathogens expressing Tet(56), a type 2 TDase^12^. Markley et al. generated several analogs of anhydrotetracycline with halogenation of the D-ring to extend the spectrum of inhibition against type 1 and type 2 TDases^19^. Liu et al. demonstrated that combining antiviral agent azidothymidine (AZT) with tigecycline decreased the survival of *E. coli* expressing Tet(X4)^33^. Xu et al. established that plumbagin, a natural naphthoquinone isolated from plants, shows synergistic effects with tetracycline antibiotics against Tet(X3)-/Tet(X4)-producing bacteria^34^. Deng et al. confirmed a combination of Bi(NO_3_)_3_ and tigecycline can prevent development of resistance in bacteria expressing Tet(X)^35^. Most recently, Williford et al. reported a series of C9-benzamide and C9-benzylamine anhydrotetracycline analogs that act as bisubstrate inhibitors of type 1 and type 2 TDases^32^. Despite these recent investigational studies, the structural characteristics of an inhibitor in clinically relevant TDases (i.e., type 1 TDases), remain unknown. Structural insights into TDase-inhibitor complexes are imperative to design potent and effective TDase inhibitors.

The structural architecture of a TDase is composed of three conserved, key features: (i) a Rossman-fold containing FAD-binding domain; (ii) a substrate-binding domain; (iii) and a C-terminal bridge helix (Fig. 1). TDases are broadly classified into two main classes: type 1 TDases (also known as Tet(X)-like^14^ TDases) and type 2 TDases (also known as soil-derived^10^ TDases). A key structural difference between type 1 and type 2 TDases is an additional C-terminal, ‘gate-keeper’ ɑ-helix present in type 2 TDases^12^ (Supplementary Fig. 2). This ‘gate-keeper’ helix regulates substrate loading and catalysis, and may plausibly clash with D-ring substituted tetracyclines that include third-generation tetracyclines. Therefore, type 2 TDases remain inactive against third-generation tetracyclines. In contrast, type 1 TDases that lack the ‘gate-keeper’ C-terminal helix can accommodate D-ring substitutions on the tetracycline moiety and inactivate all available tetracyclines. The dynamics of the bound FAD cofactor also play a critical role in enzyme catalysis/inhibition. During the substrate oxidation step of the catalytic cycle, the FAD occupies an ‘IN’ orientation to position the presumed C4a-peroxy-flavin for oxygen transfer to C11a of the bound substrate. The oxidized FAD transitions to the ‘OUT’ state for subsequent regeneration (presumably reduction by NADPH) and availability for the next round of catalysis.

**Fig. 6:**
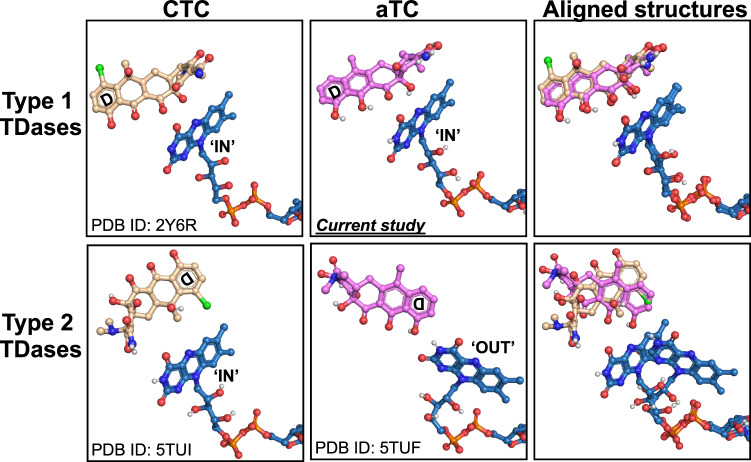
Substrate and inhibitor binding modes in type 1 and type 2 TDases. In type 1 TDases (top panels), chlortetracycline (CTC) and anhydrotetracycline (aTC) bind in a similar substrate-like orientations. The bound co-factor, FAD (shown in blue) occupies an “IN” orientation. In contrast, CTC and aTC bind in distinct orientations in type 2 TDases (bottom panels). Anhydrotetracycline (aTC) locks FAD in an inactive “OUT” orientation.

Prior to this report, no structural details have been available for type 1 TDases in complex with an inhibitor. Anhydrotetracycline is a pan-TDase inhibitor that acts against both classes of TDases in different biochemical and cellular assays^10,12,14,19^. Here, we have solved the X-ray structure of a type 1 TDase, Tet(X6), in complex with anhydrotetracycline. The Tet(X6)-anhydrotetracycline complex shows the FAD cofactor bound in an ‘IN’ conformation that is distinct from the ‘OUT’ conformation observed for FAD in X-ray structures of type 2 TDases complexed with anhydrotetracycline. The anhydrotetracycline binds in a substrate-like orientation in the Tet(X6) active site and would compete for binding with diverse tetracycline substrates (Fig. 6)^17,18^. Steady-state kinetics suggest anhydrotetracycline is a good binding ligand but a poor substrate for catalysis relative to tetracycline antibiotics. The extra site of dehydration at the C5’-C6 bond of anhydrotetracycline creates a more stable aromatic naphthalene moiety compared to the styrene moiety present in tetracyclines. Anhydrotetracycline also benefits from extended conjugation across the sensitive 1,3-diketo/enol system formed by atoms C11-C11a-C12 at the C,D-ring juncture. Oxidation at C11a of anhydrotetracycline and tetracycline presumably occurs via hydroxyl group transfer from the reactive C4a-peroxy-flavin intermediate. The extra stabilization of anhydrotetracycline is likely to reduce the nucleophilicity of the enol tautomer at C11a and slow the rate of oxidation at C11a compared to more reactive tetracycline substrates.

Type 1 TDases are more commonly found in pathogens. Despite conserved residues in the FAD-binding pocket and in substrate-binding pockets, different type 1 TDases show distinct catalytic efficiencies towards different substrates. Mutations in regions distant from ligand binding cavities may plausibly contribute to the difference in structural dynamics and influence local active site structures resulting in different catalytic efficiencies. Indeed, a surface-localized, single point mutation, Thr280Ala in Tet(X2) reduces the apparent *K*_m_ for minocycline by two-fold^36^.

Anhydrotetracycline-based inhibitors have been shown to inhibit diverse TDases in enzymatic and cellular studies^19,32^. This study provides structural insights into the binding mode of anhydrotetracycline complexed with type 1 TDase Tet(X6), and reveals key pharmacophoric features in the active site cavity, which can be explored further to develop improved anhydrotetracycline-based inhibitors with enhanced binding affinity and enzyme inhibition. Specifically, side chains of Asp61, His63, Asn112, Gln322, Glu367, Asn371 in the vicinity of the D-ring of bound anhydrotetracycline may be targeted to form additional H-bonds with the designed inhibitor through substituents on the D-ring of the anhydrotetracycline scaffold. Similarly, side chains of Asp61 and Arg213 are positioned favorably to form additional H-bonds with polar group substitutions at the C-ring of the anhydrotetracycline scaffold. Substituents on the B- and A-rings of tetracycline scaffold could possibly form additional H-bonds with the side chains of Ser238, Asn190, His234, Gln192. These findings establish a foundation for rational structure-based design of anhydrotetracycline-based inhibitors to combat antibiotic resistance conferred by type 1 TDases.

## Methods

### Cloning and protein expression

The coding region of *tet(X6)* (QHN11884.1)^20^ was cloned in a pET28 vector (cleavage sites: Age1, Kpn1) with a 6-His tag at the C-terminus. The cloned construct was then transformed into *E. coli* BL21 (DE3) to express the protein. Cells were cultured at 37 °C in LB media, containing 0.03 mg/ml kanamycin, until the OD_600_ (optical density at λ = 600 nm) reached 0.6-0.8. At that point, the temperature was lowered to 18 °C and expression was induced with 1 mM Isopropyl β-D-1-thiogalactopyranoside (Sigma-Aldrich, St. Louis, MO). The induced culture was grown overnight (~20 h) at 18 °C and centrifuged at the maximum speed (20 min, 4 °C) to pellet down the cells.

### Protein purification

The cell pellet was resuspended in the lysis buffer [50 mM Tris (pH 8.0), 100 mM NaCl, 10 mM imidazole (pH 8.0) 10 mM beta-mercaptoethanol (BME), Pierce^TM^ protease inhibitor tablet (catalog # A32963; Thermo Scientific)] and stored at −80 °C. To purify the protein, the frozen pellet was thawed in the presence of 0.25 mg/mL lysozyme. The cells were disrupted using a sonicator (ON/OFF/total-time:0.5/0.5/120 s). The cell lysate was centrifuged at 25,000 g for 20 min and the supernatant was loaded on nickel rapid run agarose beads (Goldbio) that were previously equilibrated with the wash buffer [50 mM Tris (pH 8.0), 100 mM NaCl, 10 mM imidazole (pH 8.0), 5 mM BME and Pierce^TM^ protease inhibitor tablet (catalog # A32963; Thermo Scientific). The beads were washed three times with five-column volume of wash buffer and finally eluted with 3-column volume of the elution buffer [50 mM Tris (pH 8.0), 100 mM NaCl, 500 mM imidazole (pH 8.0)]. The eluted protein sample was further purified by gel purification using HiLoad 16/600 Superdex 200 pg column (GE Healthcare) equilibrated with 10 mM Hepes (pH 7.4), 100 mM NaCl, 5 mM DTT. The fractions containing the protein of interest were pooled and concentrated using a 10 K MWCO Amicon centrifugal filter (Millipore). During all steps, the sample was kept at 4 °C.

### Crystallization, data collection, and structure refinement

Tet(X6) was concentrated to 20 mg/mL and crystallized by vapor diffusion in hanging drop at 18 °C in 0.2 M potassium thiocyanate and 20% (w/v) PEG 3350. Crystals were transferred into 0.2 M potassium thiocyanate, 20% (w/v) PEG 3350 and 20% PEG 400 for 15–30 s and flash-cooled in liquid nitrogen. 4 mM anhydrotetracycline was added to 15 mg/mL Tet(X6), centrifuged at 10,000 g for 10 min at 4 °C, and the complex crystallized by vapor diffusion in hanging drop at 18 °C in 0.2 M potassium thiocyanate and 20% (w/v) PEG 3350. The co-crystals were transferred into 0.2 M potassium thiocyanate, 20% (w/v) PEG 3350 and ethylene glycol for 15–30 s and flash-cooled in liquid nitrogen. Diffraction data were collected at 100 K on beamline 22-ID (APS). 900 frames were collected with the oscillation step of 0.2 degrees. Sample to detector distance was set to 235 mm for anhydrotetracycline-free Tet(X6) crystal and to 250 mm for Tet(X6) + anhydrotetracycline crystal. All data processing and structure analysis were performed using SBGrid^37^. Diffraction data was reduced and scaled using XDS^38^. Tet(X6) structure was solved by molecular replacement using Phaser^39^ with the Tet(X7) structure (PDB ID: 6WG9; sequence identity: ~94%) as a starting model. The protonation pattern of anhydrotetracycline was defined as previously described^40^. Structure refinement was performed in Phenix^41^ and Coot^42^. The final model was validated using the Molprobity server^43^.

### Antibiotic susceptibility and checkerboard inhibition assays

Tetracycline resistance genes were cloned into the *KpnI* and *MluI* sites of the pZE24 plasmid (Expressys); this plasmid is maintained using kanamycin and its P_lac/ara-1_ promoter can be regulated with IPTG and arabinose^44^. For high expression, 1 mM IPTG fully relieves repression by LacI^44^ thus only IPTG was used in whole-cell tests. Chemically-competent *E. coli* DH5αZ1 (Expressys) was transformed with these pZE24 constructs by heat shock. Minimum inhibitory concentrations (MICs) were measured as per Clinical and Laboratory Standards Institute (CLSI) guidelines^45^. Substrates and inhibitors were dissolved in DMSO (20 mg/mL) then diluted to working concentrations in cation-adjusted Mueller-Hinton II broth supplemented with 50 μg/mL kanamycin (Supplementary Data 2). Antibiotic susceptibility testing panels were prepared in 96-well flat-bottom microplates (Corning) by two-fold serial dilution of the antibiotic of interest (Supplementary Data 3). For checkerboard whole cell inhibition assays, anhydrotetracycline was two-fold serially diluted in a constant concentration of tetracycline (Supplementary Data 3). Liquid cultures of each strain were grown to exponential phase then diluted to a standard concentration (OD_600_ = 0.0015, which is equivalent to double ~5 × 10^5^ CFU/mL) and inoculated into each panel at a 1:1 ratio. Thus, each well had a final concentration of 50 μg/mL kanamycin, 1 mM IPTG, ~5 × 10^5^ CFU/mL (0.5 MacFarland) cells, and variable concentrations of the antibiotic of interest or anhydrotetracycline (Supplementary Data 2 and 3). Each strain-antibiotic/inhibitor combination was tested in triplicate, along with no-drug and no-cell controls. Inoculated panels were sealed with Breathe-Easy membranes (Sigma-Aldrich) and incubated at 37 °C for 20 h. MICs were scored by absorbance measurements at 600 nm (OD_600_) using the Synergy H1 microplate reader (Biotek Instruments, Inc). Synergy of inhibitor and tetracycline combinations was determined using the fractional inhibitory concentration index (FICI) method^25^ where FICI > 1 indicates antagonism, FICI = 1 indicates additivity, and FICI < 1 indicates synergy:

1 $${FICI}=\frac{{MIC}\,{A}_{{combo}}}{{MIC}\,{A}_{{alone}}}+\frac{{MIC}\,{B}_{{combo}}}{{MIC}\,{B}_{{alone}}}$$

### Characterization of substrate degradation by scanning optical absorbance spectroscopy and LC-MS

All in vitro kinetic assays were prepared open to air in non-degassed buffer solutions at room temperature. TDase reactions were prepared in 100 mM TAPS buffer (pH 8.5) with an NADPH regenerating system (40 mM glucose-6-phosphate, 4 mM NADP^+^, 1 mM MgCl_2_, 4 U/mL glucose-6-phosphate dehydrogenase), 20 μM substrate and 0.24 μM Tet(X6) (all concentrations represent final working concentrations). In vitro reactions were monitored by optical absorbance spectroscopy on an Agilent Cary 50 UV-visible spectrophotometer using polystyrene cuvettes. Reaction progress was monitored by optical absorbance spectroscopy (280−550 nm, 1 nm and 5 min intervals) over 2 h. Aliquots of reaction sample (150 μL) were removed and quenched (600 μL of 1:1 acetonitrile/0.25 M aqueous HCl) immediately after enzyme was added (0 min) and at 5, 30, 60, 90, and 120 min intervals. The quenched samples were centrifuged (5000 rpm, 4 °C) for 5 min, and 600 μL of the resulting supernatant was mixed with an Fmoc-Ala internal standard (3.12 μM final concentration) and analyzed by LC-MS in positive ion mode (single trial). LC-MS was acquired using an Agilent 6130 single quadrupole instrument (ESI + ) with G1313 autosampler, G1315 diode array detector, and 1200 series solvent module with separation on a Phenomenex Gemini C18 column, 50 × 2 mm (5 um) fit with a guard column cassette. LCMS solvents were 0.1% formic acid in H_2_O (A) and 0.1% formic acid in acetonitrile (B). Solvent gradient was linear starting from 0% B to 95% B over 20 min at a flow rate of 0.5 mL/min. LCMS data were processed using ChemStation software version B.04.02 SP1. Extracted ion chromatograms (EICs) for the expected [M + H]^+^ molecular ions corresponding to substrate and mono-hydroxylated product were normalized to the [M + H]^+^ counts for an Fmoc-Ala internal standard.

### Characterization of steady-state kinetics for substrate inactivation

All experiments were prepared open to air in non-degassed buffer solutions at room temperature. Reactions were prepared in 100 mM TAPS buffer at pH 8.5 with 0–30 µM substrate (a 65 µM concentration was included for eravacycline to reach *v*_max_), 504 µM NADPH, 5.04 mM MgCl2, and 0.4 µM Tet(X6) (final working concentrations). Reactions were initiated by the addition of Tet(X6) and were monitored continuously via optical absorbance spectroscopy at 400 nm (440 nm for aTC inactivation) for 2 min (performed in triplicate as independent trials). Initial enzyme velocities were determined by linear regression using Agilent Cary WinUV Software over the linear range of the reaction (typically between 0 to 1 min), plotted against the concentration of the substrate, and fitted to the Michaelis–Menten or allosteric sigmoidal nonlinear regression equations using GraphPad Prism 6.

### Determination of apparent Tet(X6) inhibitor IC_50_ values

All experiments were prepared open to air in non-degassed buffer solutions at room temperature. Half-maximal inhibitory concentrations (IC_50_) for the inhibition of Tet(X6) were determined from the velocities of substrate degradation in the presence of varying concentrations of inhibitor. Reaction samples were prepared in 100 mM TAPS buffer (pH 8.5) with 504 μM NADPH, 5.04 mM MgCl_2_, 25.3 μM TC, varying concentrations of inhibitor (typically 0–146 μΜ), and 0.4 μM Tet(X6) (final working concentrations). Reactions were initiated by the addition of Tet(X6) and were monitored continuously via optical absorbance spectroscopy at 400 nm for 2 min (performed in triplicate as independent trials). Initial enzyme velocities were determined by linear regression using Agilent Cary WinUV Software over the linear range of the reaction (typically between 0 to 1 min). The velocities were plotted against the logarithm of inhibitor concentration, and apparent IC_50_ values were determined using nonlinear regression analysis in GraphPad Prism v6. Each set of experiments included a no-TDase control reaction which was used as the full enzyme inhibition velocity and assigned to an inhibitor concentration of 1 × 10^15^, as well as a no-inhibitor control which was assigned an inhibitor concentration of 1 × 10^–15^. A no-TC control was also performed to search for potentially competitive background signals generated from the enzymatic degradation of the inhibitor itself. For all inhibitor-enzyme combinations, the initial velocities of the no-TC controls were negligible.

### Statistics and reproducibility

X-ray structural analysis statistics including number of crystals used per data set are include in Table 1. Standard statistical analysis for X-ray diffraction data processing and analysis were adhered to and presented in Table 1. Enzyme assays were performed in two or three independent trials and whole cell inhibition assays were performed in triplicate.

### Reporting summary

Further information on research design is available in the Nature Portfolio Reporting Summary linked to this article.

## Supplementary information

Supplementary Information-New

Description of Additional Supplementary Files

Supplementary Data 1

Supplementary Data 2

Supplementary Data 3

Supplementary Data 4

Reporting Summary

## Data Availability

Atomic coordinates and structure factors have been deposited in the Protein Data Bank with the accession codes 8ER1 and 8ER0. All other data generated or analyzed during this study are included in this published article and its supplementary information files. Source data for figures can be found in Supplementary Data 4.
