# Publisher Correction: Structure of anhydrotetracycline-bound Tet(X6) reveals the mechanism for inhibition of type 1 tetracycline destructases

**DOI:** 10.1038/s42003-023-04906-y

**Published:** 2023-05-11

**Authors:** Hirdesh Kumar, Emily E. Williford, Kevin S. Blake, Brett Virgin-Downey, Gautam Dantas, Timothy A. Wencewicz, Niraj H. Tolia

**Affiliations:** 1 Host-pathogen interaction and structural vaccinology section (HPISV), National Institute of Allergy and Infectious Diseases (NIAID), National Institutes of Health (NIH), Bethesda, MD USA; 2 Department of Chemistry, Washington University in St. Louis, One Brookings Drive, St. Louis, MO 63130 USA; 3 The Edison Family Center for Genome Sciences and Systems Biology, Washington University School of Medicine, St. Louis, MO USA; 4 Department of Pathology and Immunology, Division of Laboratory and Genomic Medicine, Washington University School of Medicine, St. Louis, MO USA; 5 Department of Molecular Microbiology, Washington University School of Medicine, St. Louis, MO USA; 6 Department of Biomedical Engineering, Washington University in St. Louis, St. Louis, MO USA; 7 Department of Pediatrics, Washington University School of Medicine, St. Louis, MO USA

**Keywords:** X-ray crystallography, Bacterial structural biology

Correction to: *Communications Biology* 10.1038/s42003-023-04792-4, published online 17 April 2023.

The PDF version of the Article contained a duplication of Figure 1 at the position of Figure 5. This has now been corrected in the PDF; the HTML version of the Article was correct from the time of publication.

