# Supplementary material for: Structure of anhydrotetracycline-bound Tet(X6) reveals the mechanism for inhibition of type 1 tetracycline destructases
Source: Commun Biol. 2023 Apr 17;6:423. doi: 10.1038/s42003-023-04792-4 (PMC10106456; doi:10.1038/s42003-023-04792-4)
Supplement: Supplementary file 1 — Supplementary Information-New [file 42003_2023_4792_MOESM1_ESM.pdf]

a

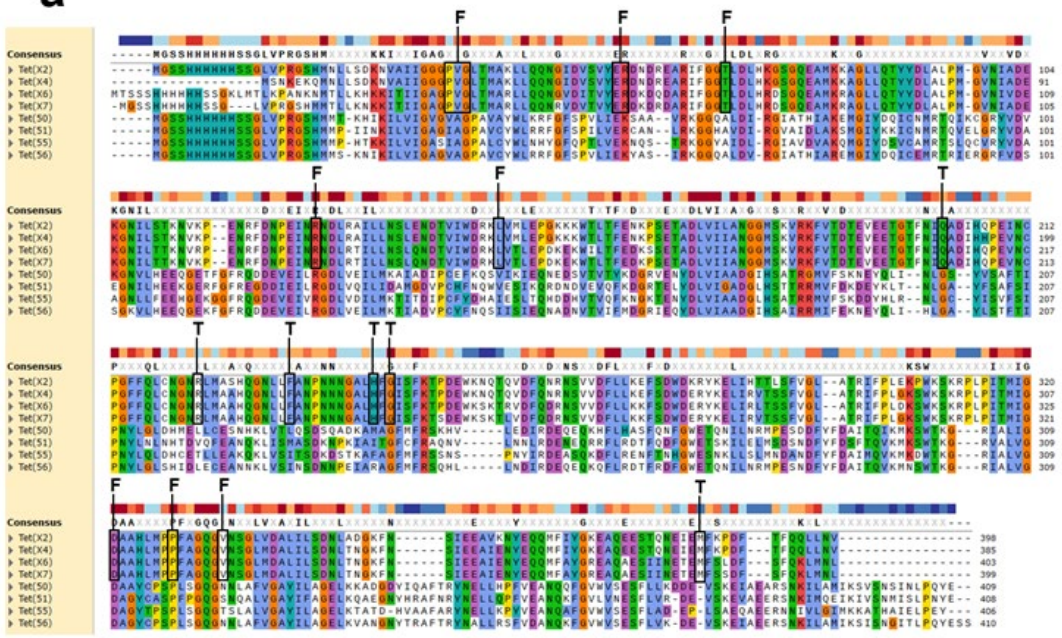

b

		Type 1				Type 2				
		X2	X4	X6	X7	50	51	55	56	
Type 1	X2		0.4 (317)	0.4 (336)	0.3 (320)	3.4 (220)	5.1 (268)	3.3 (244)	3.3 (207)	r.m.s.d. (in Å)
	X4	94.3		0.5 (321)	0.4 (313)	3.2 (215)	4.4 (248)	3.3 (258)	4.3 (241)	
	X6	81.2	86.0		0.3 (309)	2.5 (226)	4.9 (257)	3 (242)	3.8 (251)	
	X7	82.3	87.3	94.2		2.7 (243)	4.3 (250)	3.1 (253)	3.7 (241)	
Type 2	50	25.2	20.4	22.3	21.7		2.1 (346)	0.7 (318)	1.9 (346)	
	51	25.0	21.0	23.7	22.8	65.2		1.9 (338)	0.7 (320)	
	55	26.1	21.3	23.0	22.3	62.6	61.3		1.4 (306)	
	56	23.4	19.1	21.1	21.0	79.4	67.9	62.3		
		Sequence identity (%)								

Supplementary figure 1: A comparison of different TDase structures. (a) Multiple sequence alignment of different TDases sequences. Please note that protein sequences were retrieved from RCSB database in fasta format. Clustal omega was used to perform multiple sequence alignment. FAD-interacting residues are labeled as “F” and tetracycline substrate-interacting residues are labeled as “T”. PDB codes for different structures are as follows Tet(X2)-4A6N, Tet(X4)-7EPW, Tet(X6)-8ER1, Tet(X7)-6WG9, Tet(50)-5TUF, Tet(51)-5TUK, Tet(55)-5TUL, Tet(56)-5TUM. (b) Percentage sequence identity (lower half matrix; shown in orange color) and Cα-based r.m.s.d of different TDase structures. Total number of superimposed Cα atoms for each alignment are shown in parentheses (upper half matrix; shown in blue color).

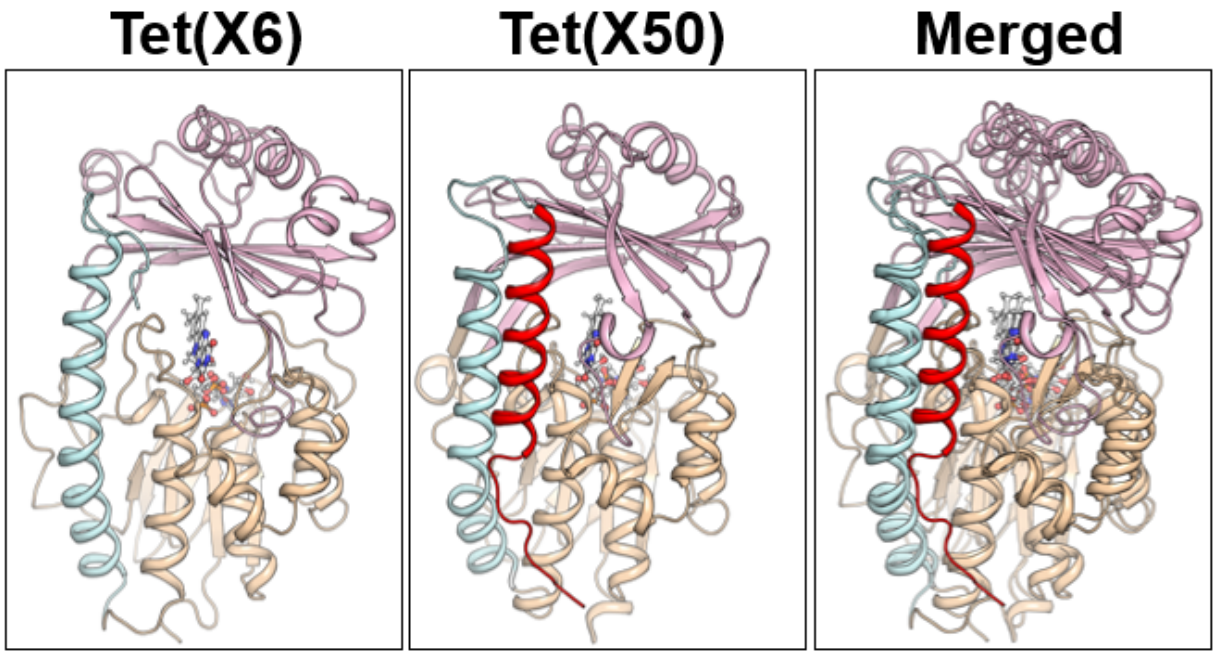

Supplementary figure 2: Structural comparison of Tet(X6) (a type 1 TDases; PDB ID: 8ER1) and Tet(50 (a type2 TDase): Tet(50) (PDB ID 5TUF, chain A). Substrate-binding domain is colored pink, FAD-binding domain is colored orange. The C-terminal bridge helix is colored blue. Please note Tet(50) contains an additional 'gate-keeper' α-helix at the C-terminus (shown in red). This α-helix is a characteristic of type 2 TDases and is absent in type 1 TDase structures.

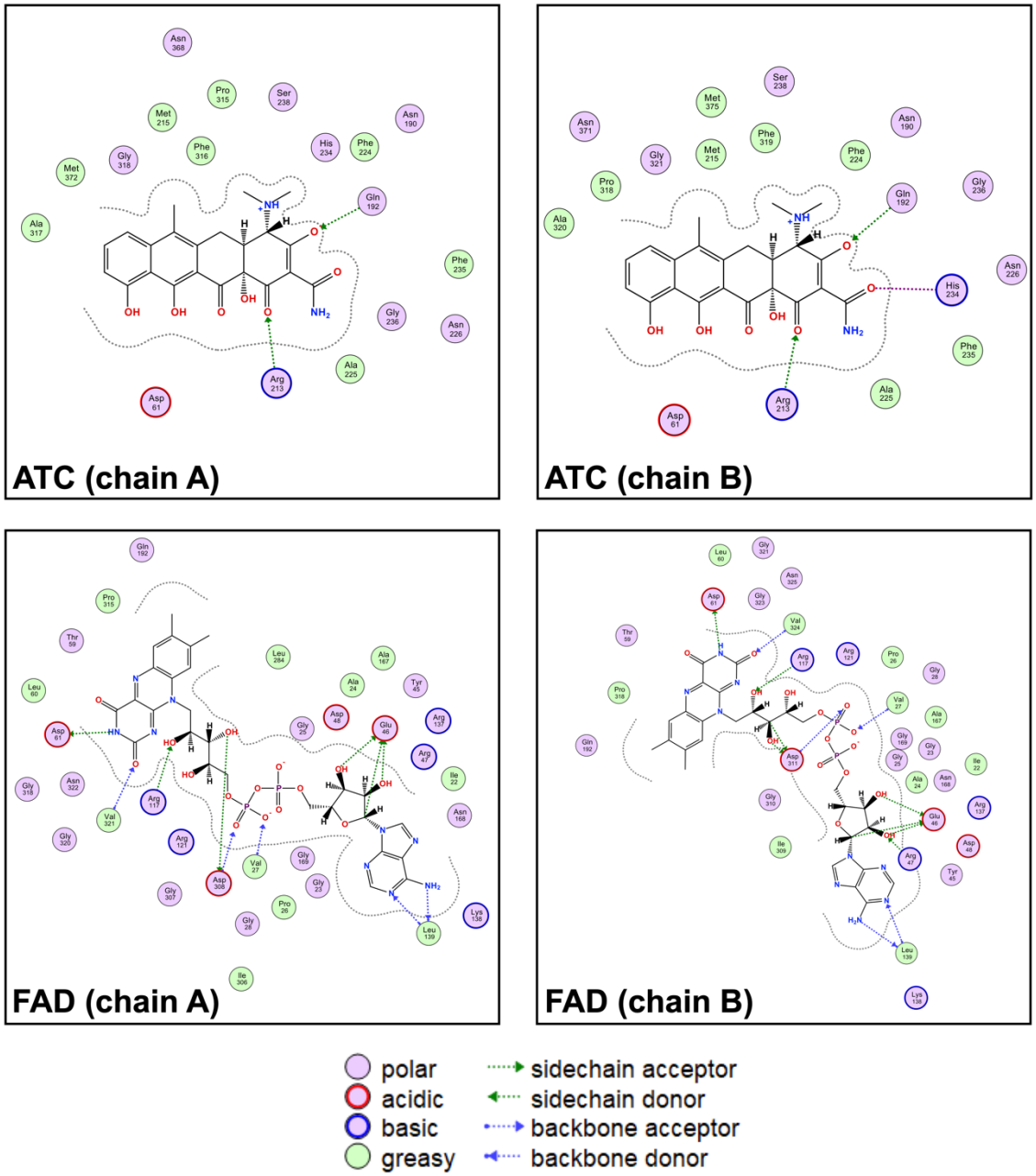

Supplementary figure 3: Two-dimensional ligand interactions diagram of ATC and FAD interactions in the two chains of the anhydrotetracycline bound Tet(X6) structure determined here (8ER0). Residues are color-coded as per their physico-chemical properties. H-bonds are shown in dotted arrows.

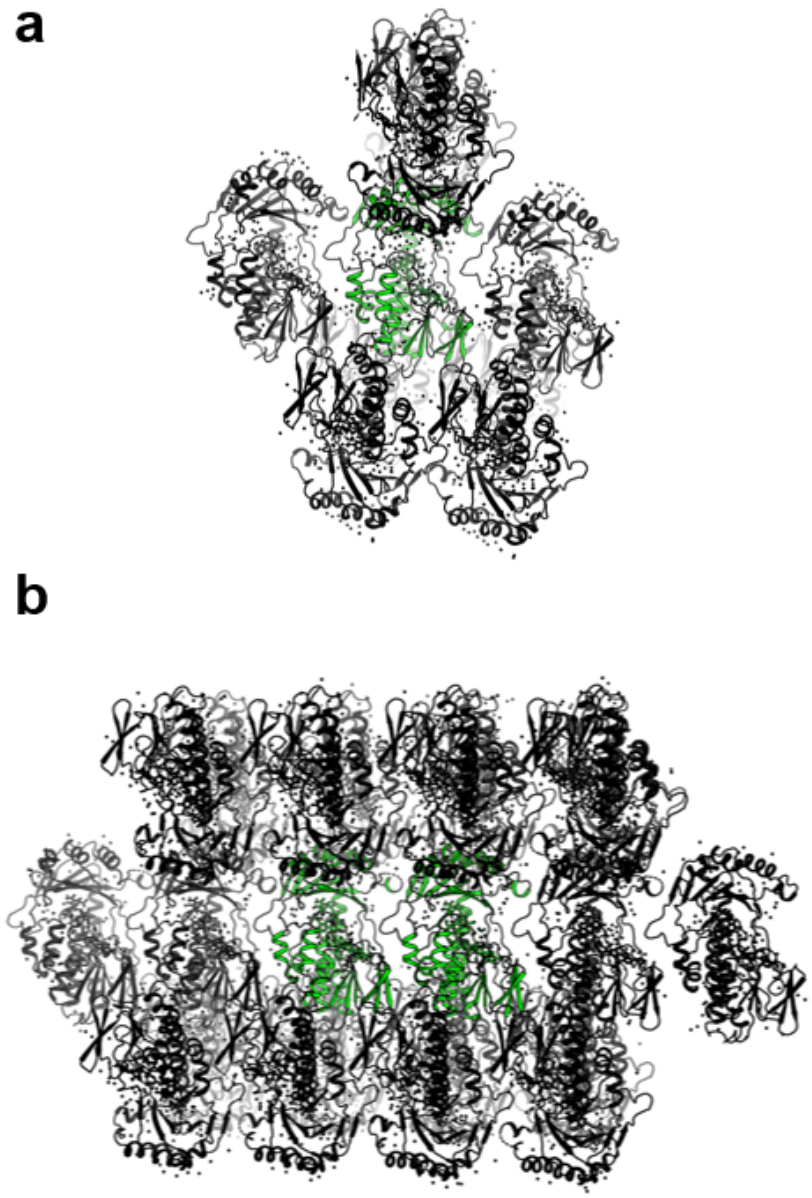

Supplementary figure 4: Crystal packing of the two Tet(X6) X-ray crystal structures. (a) Original anhydrotetracycline-free Tet(X6) structure (PDB ID: 8ER1) is show in green and full view (zoomed out) contains additional 5Å symmetry mates (in black). (b) Original anhydrotetracycline-bound Tet(X6) structure (PDB ID: 8ER0) is shown in green and full view (zoomed out) contains additional 5Å symmetry mates (in black).

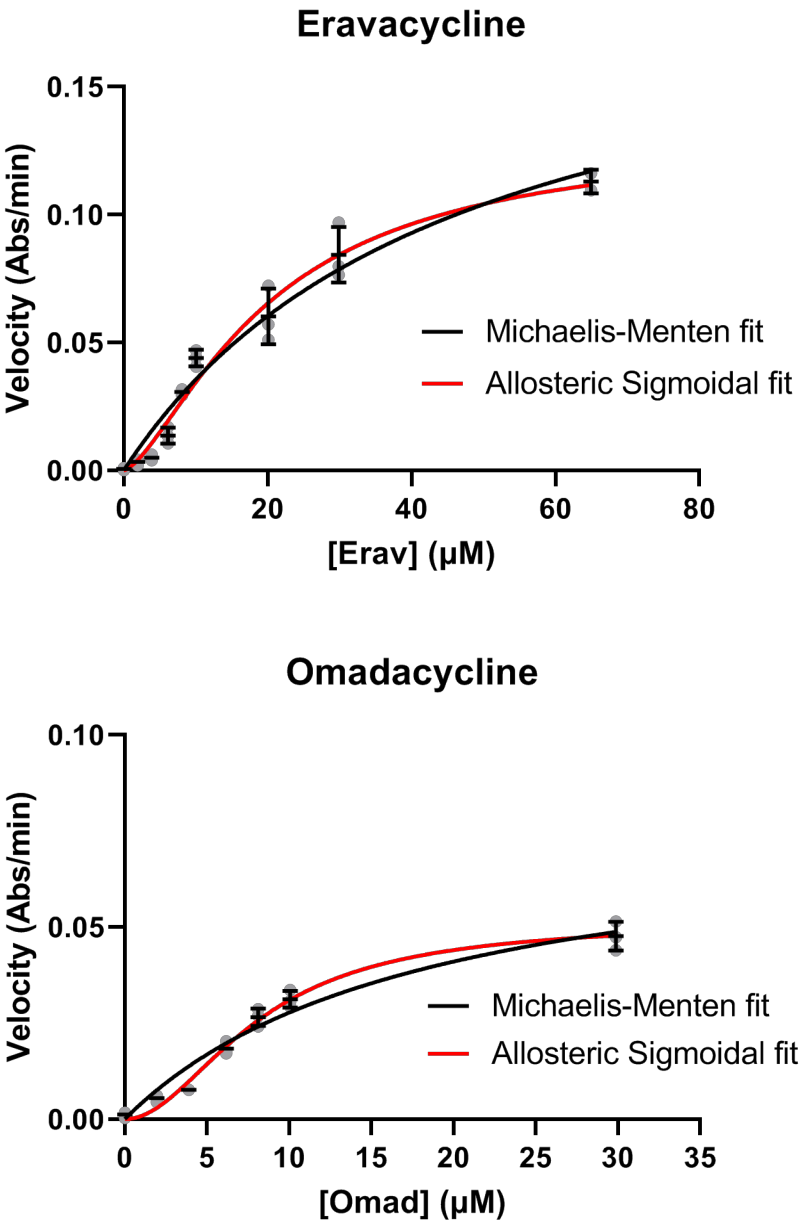

Supplementary figure 5: Comparison of Michaelis-Menten and allosteric sigmoidal data fits for Tet(X6)-catalyzed degradation of eravacycline and omadacycline under steady-state conditions. Velocity on the y-axis represents absorbance per minute measured continuously at 400 nm. The x-axis represents the working concentration of substrate, eravacycline or omadacycline, in micromolar. Parameters and goodness of fit for each method are provided in Supplementary Table 2. The Michaelis-Menten curves for these plots are identical to the Michaelis-Menten curves used in Figure 2, panel (c) of the main text. Error bars represent standard deviations for three independent trials.

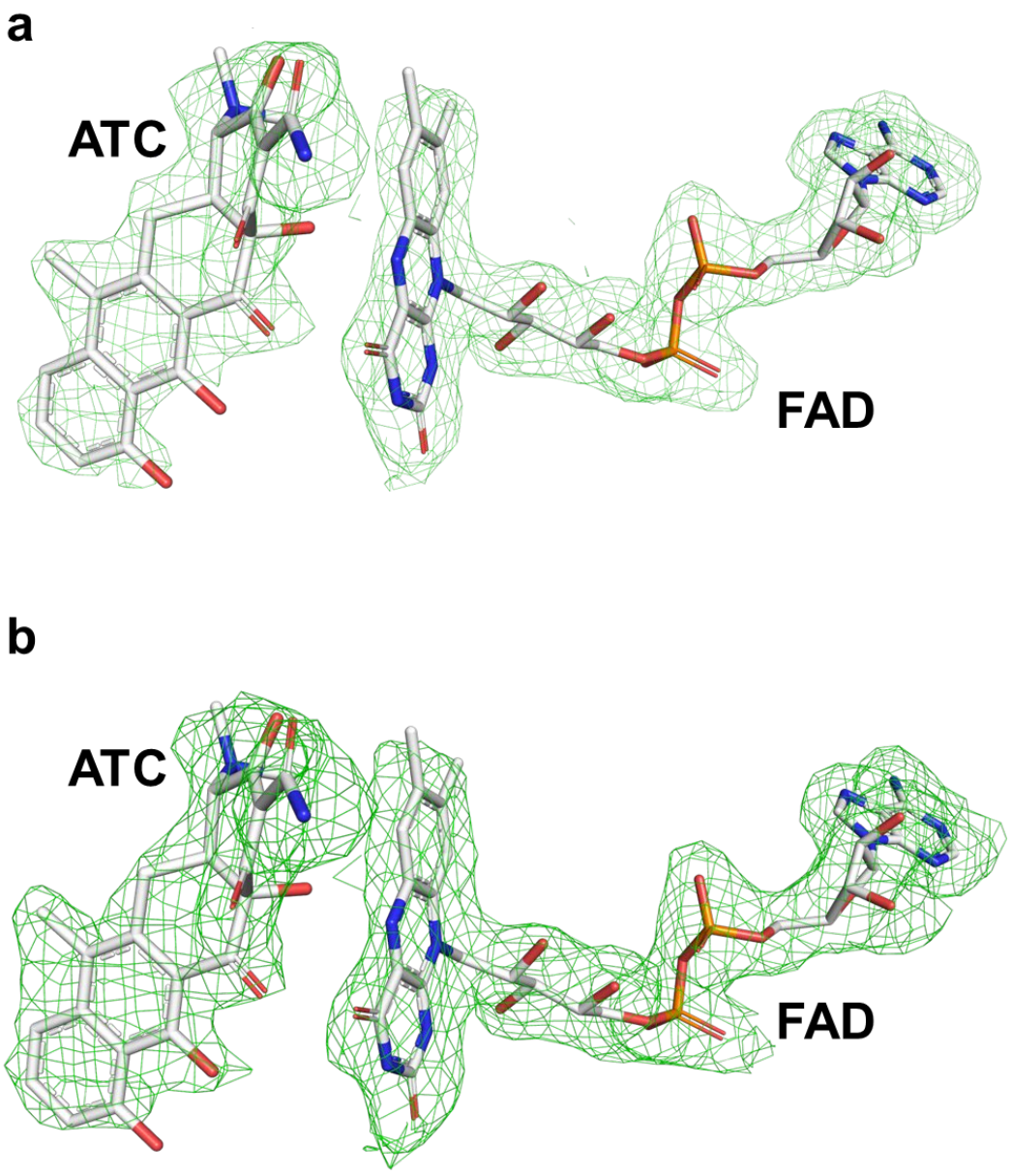

Supplementary figure 6: Polder maps (Fo-Fc map, σ = 3) of bound anhydrotetracycline (ATC) and FAD in the two chains are shown (PDB ID: 8ER0). (a) Polder map for ATC and FAD in chain A. (b) Polder map for ATC and FAD in chain B.
