# Supplementary material for: Structure of anhydrotetracycline-bound Tet(X6) reveals the mechanism for inhibition of type 1 tetracycline destructases
Source: Commun Biol. 2023 Apr 17;6:423. doi: 10.1038/s42003-023-04792-4 (PMC10106456; doi:10.1038/s42003-023-04792-4)
Supplement: Supplementary file 2 — Description of Additional Supplementary Files [file 42003_2023_4792_MOESM2_ESM.pdf]

Description of Additional Supplementary Files

File name: Supplementary Data 1

Description: Curve fitting to Michaelis-Menten and allosteric sigmoidal models.

File name: Supplementary Data 2

Description: AST panel concentrations.

File name: Supplementary Data 3

Description: Checkerboard panel concentrations.

File name: Supplementary Data 4

Description: The source data behind the graphs in the paper.
